# Bibliometric-Based Evaluation of the Neuromarketing Research Trend: 2010–2021

**DOI:** 10.3389/fpsyg.2022.872468

**Published:** 2022-08-02

**Authors:** Zeren Zhu, Yuanqing Jin, Yushun Su, Kan Jia, Chien-Liang Lin, Xiaoxin Liu

**Affiliations:** ^1^College of Science and Technology, Ningbo University, Ningbo, China; ^2^Research Center for Ningbo Bay Area Development, Ningbo University, Ningbo, China; ^3^School of Public Administration, Zhejiang University of Technology, Hangzhou, China

**Keywords:** neuromarketing, bibliometric, neuroscience, citespace, marketing

## Abstract

Neuromarketing has become a new and important topic in the field of marketing in recent years. Consumer behavior research has received increasing attention. In the past decade, the importance of marketing has also been recognized in many fields such as consumer behavior, advertising, information systems, and e-commerce. Neuromarketing uses neurological methods to determine the driving forces behind consumers’ choices. Various neuroscience tools, such as eye movements, have been adopted to help reveal how consumers react to particular advertisements or objects. This information can be used as the basis for new advertising campaigns and brand promotions. To effectively explore the research trends in this field, we must understand the current situation of neuromarketing. A systematic bibliometric analysis can solve this problem by providing publishing trends and information on various topics. In this study, journals that focused on neuromarketing in the field of marketing between 2010 and 2021 were analyzed. These journals were core journals rated by the Association of Business Schools with three or more stars. According to the data analysis results, neuromarketing has 15 main journals with relevant papers. Based on the data collected by the Web of Science (WOS), this study mainly collected 119 references and analyzed the most productive countries, universities, authors, journals, and prolific publications in the field of neuromarketing *via* Citespace. Through the analysis of knowledge maps, this study explored the mapping of co-citation, bibliographic coupling (BC), and co-occurrence (CC). Moreover, the strongest citation bursts were used to study popular research at different time stages and analyze the research trends of neuromarketing research methods and tools. This study provides an overview of the trends and paths in neuromarketing, which can help researchers understand global trends and future research directions.

## Introduction

The neuromarketing field has grown exponentially in recent years, and studies in marketing academic journals using neuroscience methods have increased significantly (de Oliveira and Giraldi, 2017; Lee et al., 2018). In the early period, it was controversial among researchers whether this mixed field was beneficial to its parent disciplines (consumer psychology and neuroscience) and how these research results would be reintegrated into these disciplines (Plassmann et al., 2012; Fortunato et al., 2014; Rawnaque et al., 2020). Moreover, most reviewed papers were from lower-ranking journals, and in early studies, different researchers understood the concept of “neuromarketing” differently. This created a lack of clear guidance regarding positives and negatives in defining neuromarketing research (Plassmann et al., 2012; Lee et al., 2018). Therefore, Plassmann et al. (2012) put forward opinions on the analytical direction of the definition of future research to promote the development of guided studies on concept definition and research classification. de Oliveira and Giraldi (2017) analyzed and collated the definitions of neuromarketing in previous studies and summarized and provided a more accurate definition of neuromarketing.

Neuromarketing uses the non-invasive brain signal recording technology to directly obtain consumers’ feedback on marketing stimuli, instead of traditional investigation methods (Fortunato et al., 2014; Lee et al., 2017; Nilashi et al., 2020; Rawnaque et al., 2020). These technologies are used to study attention, emotional memory, and user experiences in the field of advertising (Bakalash and Riemer, 2013; Adil et al., 2018; Clark et al., 2018). Simultaneously, several studies on the feasibility of applying new equipment and methods in this field are constantly emerging (Venkatraman et al., 2012; Zhang et al., 2020). While brain data can be used to predict consumer behavior (e.g., Knutson and Genevsky, 2018; Krampe et al., 2018; Motoki et al., 2020; Tong et al., 2020), compared to traditional marketing measurement methods, the application of neuroscience is more scientific in predicting consumers’ marketing behavior (Berkman and Falk, 2013; Smidts et al., 2014; Lee et al., 2018). Examples include the advertising effect (Ramsøy, 2019; Grigsby and Mellema, 2020) and purchasing behavior (Çakir et al., 2018; Kim and Lakshmanan, 2021). However, as traditional measurement methods are only used to understand cognitive behavior subjectively, there are still weaknesses in many behavior predictions. However, predictions made by combining neuroscience with traditional measurement methods, and analyzing brain activity through neuroscience, can explain consumer behavior more effectively (Motoki et al., 2020).

As there are many branches of research concepts in neuroscience, and marketing is a branch of neuroscience, researchers often lack the knowledge to conduct research in this field. Therefore, the effective systematic induction and summary of neuromarketing research are of great significance for researchers who have newly entered the research field or wish to engage in related studies. In developing the trend of neuroscience, Yeung et al. (2017) applied bibliometrics and Bradford’s law to explain research development in neuroscience. The core cited literature originated from a few core journals, which also advanced a specific discussion on the overall trend of neuroscience. Later, Kocak et al. (2019) discussed the trend analysis of neuroscience research by Turkish research institutions and authors in the top journal, *Scientometrics*, in the bibliometric analysis field. They subsequently proposed the main research directions and future research threads *via* cluster analysis and atlas. In the management and entrepreneurship field, Cucino et al. (2021) also conducted a relevant literature analysis. Their research results established five future research topics, namely, the cultivation of dynamic capability of entrepreneurs in the process of innovation and development, the development of leadership, the construction process of leadership, the perspective of leadership in biology, and the application of neuroscience in the ecosystem. The abovementioned neuroscience research demonstrates that there are no relevant studies on neuromarketing. Therefore, through a summary of core journals, researchers who have a preliminary understanding of neuromarketing can learn about the research and development status of this field and gaining entry to this field.

For the literature analysis of neuromarketing in this study, CiteSpace was primarily used as a tool for bibliometric analysis. The visualization software was used to analyze literature groups and data. This can highlight potentially important patterns and trends, and the theory of scientific changes can guide the exploration and visualization of knowledge structures and the interpretation of dynamic patterns (Chen, 2017). Among the data analysis software commonly used in bibliometrics, such as VOS Viewer, SALSA, and PRISMA, CiteSpace is user-friendly, can generate a burst detection algorithm and time zone view according to the time change, and can conduct research trend prediction and related exploration of hot spot mutations (Xu et al., 2021). In this study, bibliometrics was used to study and analyze the research process and future development trends of this field using the CiteSpace software. We hoped to address the following problems: (1) understanding the current research situation in neuromarketing, including the source areas and authors of main articles, high-frequency keywords, and keyword evolution; and (2) the research methods, experimental methods, and research focus used in neuromarketing in recent years.

## Literature Review

The word “neuromarketing” was first proposed in June 2002 by an Atlanta advertising company in the United States. In its report, a new department conducted marketing research using functional magnetic resonance imaging (fMRI) (Fisher et al., 2010). Lee et al. (2007) defined neuromarketing as the application of neuroscience methods, including psychophysics and direct brain activity, to analyze and understand human behaviors related to marketing practice. Therefore, neuromarketing is an interdisciplinary research field. It uses various tools traditionally used for neural feedback, biofeedback, and metabolic process measurement in medicine, psychiatry, and psychology, combined with traditional marketing tools. It aims to elucidate the reactions of conscious and unconscious economic agents of the most diverse emotional, cognitive, physiological, and psychological types, and the behaviors and thoughts related to typical problems in marketing and its various subfields (de Oliveira and Giraldi, 2017).

The neuromarketing technology has been used to explore consumer preferences (Murphy et al., 2008), which has aroused a great interest in marketing research companies and has also created discomfort in some individuals. However, this has not thwarted the curiosity of academic researchers (Lee et al., 2007; Murphy et al., 2008). In neuromarketing, non-invasive brain signal recording techniques are used to directly obtain consumer feedback on marketing stimuli to replace traditional investigation methods (Fortunato et al., 2014; Lee et al., 2017; Nilashi et al., 2020; Rawnaque et al., 2020). These techniques include fMRI, positron emission tomography (PET), magnetoencephalography (MEG), transcranial magnetic stimulator (TMS), electroencephalogram (EEG), galvanic skin response (GSR), and eye tracking. These techniques are used to study attention, emotional memory, and user experience in advertisements (Bakalash and Riemer, 2013; Adil et al., 2018; Clark et al., 2018). For example, Krampe et al. (2018) used fNIRS to study consumers’ neural responses to different marketing communication strategies at the point of sale (PoS) and proved that the experimental results were consistent with those of previous studies using other methods. Çakir et al. (2018) studied the development of a neurophysiological information-purchasing behavior model based on fNIRS measurements. Cerf et al. studied the possibility of using single-neuron recordings in research on marketing and consumer-related fields.

In this study, the core journals in marketing were selected for literature analysis. We aimed to elucidate the development trend of this field by analyzing related studies on neuromarketing, to promote researchers’ understanding of this topic, and to provide pathways for future research.

## Dataset and Research Methodology

### Dataset

All articles in this study were obtained from the citation databases of the Science Citation Index (SCI) and Social Science Citation Index (SSCI) and were obtained from the Web of Science (WOS) created by the Institute of Science Information (ISI). Thus, high-quality literature datasets were provided, which can be used in bibliometric research and scientific research (Vílchez-Román et al., 2020; Su et al., 2021; Tang et al., 2021; Jia et al., 2022). Despite the growing importance of neuroscience in the field of marketing, to date, less research has been conducted on the application of bibliometric methods in the field. Moreover, only Barros et al. (2018) discussed the topic of trend analysis in the literature related to neuromarketing between 2010 and 2016. However, the duration of his study and the scope of his analysis were relatively short. Therefore, this study aimed to expand the scope and duration of and provide a more in-depth analysis of trends in neuromarketing. In addition, a search of the WOS for relevant keywords revealed that the number of core journals issued before 2010 was low and only increased significantly from 2009. We also selected 2010 for the literature analysis in this study because there was only one highly cited paper before 2009 for several years (Wedel and Pieters, 2000; Pieters and Wedel, 2004; Chandon et al., 2009). However, from 2010 onward, it is likely that there would be several highly cited papers annually (Pieters et al., 2010; Reimann et al., 2010). In summary, 2010 was used as the data selection criterion in this study. The retrieval period was from 2010 to 2021 to ensure the rationality and importance of the retrieved data. This study mainly referred to the Chartered Association of Business Schools, a joint organization of British business schools that publishes the Academic Journal Guide every 3 years. The data acquisition mainly referred to the benchmark obtained by taking the journals rated above three stars in marketing in 2021 as samples; there were 20 major marketing journals in total. In the method of selecting keywords, this study focused directly on marketing journals. Therefore, the following retrieval methods were used for the keywords: TS = [(“FMRI” OR functional magnetic resonance imaging) OR (eye tracking) OR (event-related) OR (electroencephalography OR “EEG”) OR (eye fixation related potential OR “EFRP”) or (neuroscience) OR (Neuromarketing OR Neuro-marketing)]. Finally, 126 papers were preliminarily obtained.

However, some problems remained regarding the papers obtained through WOS. For example, the subject and keywords described the text; however, they were not directly related. Therefore, follow-up data cleaning can improve the quality of samples and the reliability of bibliometric analysis results (Cobo et al., 2011). However, data cleaning cannot be judged by other analysis tools or keywords and must be filtered manually. Therefore, in this study, based on the practices of Wang and Ngai (2020) and Jia et al. (2022), the collected samples were filtered manually. In filtering, two assistant university professors and two researchers of this study jointly read the abstract of the paper and confirmed whether the research topic and content were consistent with this research topic in multiple ways. If there was no relevance, then the articles were deleted. Following the above process, seven articles were deleted, and 119 articles were consistent with the topic direction. Reasons for deletion included the research content being contrary to this study and the research type coinciding with this study.

### Research Methodology

A visual atlas reflects the knowledge network in a specific research field, through which the research status and hotspots can be understood, and the research frontier in the subject field can be explored (Chang, 2017; Muñoz-Leiva et al., 2021). The information visualization software (CiteSpace 5.7) developed by Professor Chen Chaomei of Drexel University in the United States was used to draw the knowledge map of the neuroscience research literature. In the CiteSpace interface, the time span was set from 2000 to 2021, the time slice was set to 1, the node types were author, institution, and keyword, respectively, and the selection criteria were G-index, with k = 25. The neuroscience research literature was analyzed using the knowledge map obtained in this study, and the process framework of this study was organized, as shown in Supplementary Material 1.

## Results

### Publication Trends

**FIGURE 1:**
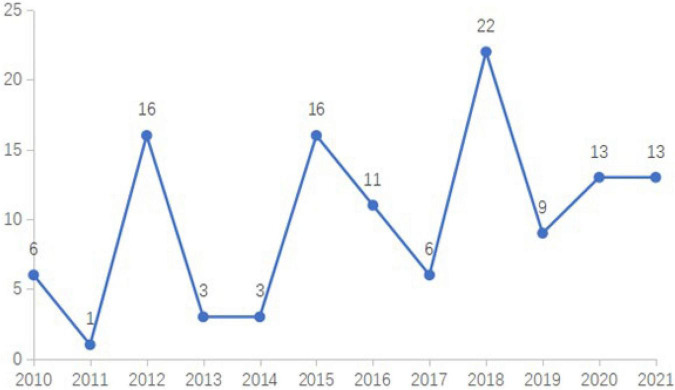
Annual publishing: 2010–2021.

The period was limited to 2010–2021, and the article type was limited to Article. Finally, 119 papers met the inclusion criteria. The annual number of publications is shown in Figure 1. The annual publication number of journals reflects the research and development levels of this discipline. The publication status from 2010 to 2021 indicates that more than one paper was published on this topic each year and that neuromarketing was still emerging. According to the summary of publication numbers, there were three periods when neuroscience research was published more prominently, namely, in 2012, 2015, and 2018, mainly due to special issue calls for papers. In 2012, 12 studies on neuroscience were collected in the special issue of the *Journal of Consumer Psychology*, under the title, “Brand Insights from Psychological and Neurophysiological Perspectives.” In 2015, 10 articles were collected in the special issue of the *Journal of Marketing Research*, with the topic of “Neuroscience and Marketing.” In 2018, nine articles were collected in the special issue of the *European Journal of Marketing*, with the topic of “Neuromarketing.” However, since 2016, neuromarketing research has shown steady growth. Specifically, 13 papers were retrieved and published by the end of 2021, indicating that the mixed discipline of neuroscience and marketing has become a popular topic and research frontier in marketing.

According to 119 articles in neuromarketing obtained from WOS data (refer to Table 1), the statistical description and analysis of the publication number of journals can reflect the development of subject knowledge structure in neuromarketing and provide guidance for later researchers to submit relevant papers. The *Journal of Marketing Research* has contributed the most to the field of neuromarketing. A total of 22 pieces of literature were published, with a total of 1,106 citations, and a citation rate of 50.27 per article. Most works focused on the relationship between the preference of consumer decision-making behavior and marketing choice and model, while others focused on the assessment of the advertisement model and the benefit of information search for the market. The *Journal of Advertising Research* ranked second in publication numbers, with a cumulative citation rate of 191 and an average citation rate of 11.24. The *European Journal of Marketing* ranked third, with a publication number of 15 articles. Compared to the second-ranking journal, its cumulative citation rate of 191 was identical; however, the average citation rate of 12.73 was higher. In particular, the *Journal of Consumer Psychology* contained only 13 articles, but its average citation rate was the highest among all journals, totaling 80.54. According to the relevant articles, among the top six papers cited in marketing from 2010 to 2021, three were from this journal [Schmitt (2012); Reimann et al. (2010), and Milosavljevic et al. (2012)]. It can be inferred that the *Journal of Consumer Psychology* was the core reference journal for most researchers studying neuromarketing-related issues.

**TABLE 1:** Summary of journals details.

Rank	Journals	Documents	TC	D|TC
1	Journal of marketing research	22	1106	50.27
2	Journal of advertising research	17	191	11.24
3	European journal of marketing	15	191	12.73
4	Journal of consumer psychology	13	1047	80.54
5	Journal of advertising	11	172	15.64
6	Journal of interactive marketing	9	111	12.33
7	Journal of consumer research	7	332	47.43
8	Marketing letters	6	115	19.17
9	Marketing science	5	139	27.80
10	Journal of marketing	3	273	91.00
11	Journal of the academy of marketing science	3	62	20.67
12	Industrial marketing management	3	9	3.00
13	Journal of retailing	2	133	66.50
14	International journal of research in marketing	2	26	13.00
15	Marketing theory	1	6	6.00

*TC, total citations; D|TC, average number of citations per article.*

### Author’s Cooperation Network

The authors’ co-occurrence (CC) had 214 nodes and 307 connections in the map, and the network density was 0.0135. This indicates that the cooperative network density between the authors is low, the author’s cooperative relationship is not close enough, and the research authors are relatively scattered. Price’s law suggests that only core authors post more than 50% of the total collaboration in the field (Price, 1963), which proves that authors have a strong cooperative relationship. According to Price’s law, the M-value of the core author’s post volume is 1.498. In this study, we set two posted articles as the criteria for viewing an author as the core author. In this study, 55 of the 76 core authors accounted for 46.22% of the 119 posts, indicating that the authors did not cooperate sufficiently. As shown in Supplementary Materials 2, 3, the cooperation of existing authors is also mainly based on external cooperation. For example, for Reimann, Martin, Bechara, and Antoine, all from the University of Southern California, the published relationship between them is weak. Other authors with more published articles have a wide range of institutions.

According to the authors and organizations with more than three publications, Supplementary Material 3 shows that there are nine scholars with more than three publications, accounting for 4.20% of the total number of scholars. The highest number of publications was four, while authors with one publication accounted for 64.49%. Comparing Lotka’s law in the three Bibliometrics laws, the total number of authors of n papers was one-nth of the total number of authors of a published paper, that is, the inverse square law of scientific productivity. Moreover, the total number of authors who published only one paper was approximately 60% of all authors (Lotka, 1926; Su et al., 2020). Therefore, the publication number of this study accorded with the concept of Lotka’s law, and most researchers published only one study. This shows that there were few outstanding high-yield core authors in the field of “neuroscience,” and most scholars were new to the field. Although there were no scholars with a high number of publications in this field, regarding the cited frequency, the article published by Venkatraman et al. (2015) was the most cited paper for studying neuromarketing (the top 1% cited globally), with a cited frequency of 191. Therefore, there is an opportunity for the continuous development of neuromarketing.

Regarding the citation rate of authors, according to the WOS database analysis, there were six pieces of literature with a high citation rate (>150). Among these, the most cited paper was conducted by Venkatraman et al. (2015), which was the only study with a high citation rate. In this study, a neurophysiological method was used to assess consumers’ response evaluation to TV advertisements. Traditional self-reports, implicit measures, eye tracking, biometrics, electroencephalography, and fMRI were used to make predictions to determine the influencing factors of advertisement benefit evaluation. In addition, five other pieces of literature had high citation rates, and the main research topics were neural measurements combined with consumers’ reactions to explore what factors can better influence consumers’ visual attention (Atalay et al., 2012; Milosavljevic et al., 2012). Notably, Pieters et al. (2010) have the highest current citation rate. This may be related to their research content, and it is essential to eliminate the interference of advertisement complexity in the follow-up experiments. Similarly, Schmitt’s (2012) model can be integrated from empirical research on brand consumers’ psychology and personal construction (e.g., brand classification, emotion, personality, symbol, and attachment). Unlike most studies, Reimann et al. (2010) explored the factors influencing consumers’ decision-making from the perspective of package design, rather than the mainstream advertisement communication model itself. This also provides an interesting guide for follow-up research (refer to Supplementary Material 4).

### Countries and Institutions

Analyzing publishing organizations can reflect high-yield research institutions and cooperation in this field. The CC atlas of publishing organizations was drawn using CiteSpace, and statistical information on the publishing situation of research institutions was obtained. According to Price’s law, the value of M was calculated to be 2.81, and the integer was 3. That is, research institutions with more than three publications were regarded as core research institutions. The research institutions with the highest number of publications were Michigan University (11 articles), Erasmus University Rotterdam (8 articles), Duke University (6 articles), and other universities, indicating that these organizations played a key role in neuroscience research. Regarding the types of research institutions, nine universities had published more than five articles among 218 universities or research institutions. This indicates that neuromarketing research was not being conducted by a few universities; moreover, it had spread gradually. Geographically, the research institutions were mainly distributed in developed countries, such as the United States and the Netherlands, which is closely related to the degree of neuroscientific development in those countries. There were 170 nodes and 221 connections in the CC atlas of publishing organizations, and the network density was 0.0154. Most nodes were distributed sporadically, and the connections between nodes were few and thin, which indicates that research institutions were scattered, except for the United States. Moreover, cooperative research results were few, and an academic research team with mutual integration and development had not yet been formed, which also reflected the dominant position of the United States in neuroscience. The existing cooperation among research institutions has mainly focused on the close cooperation of several universities. Examples include cooperation between Michigan University and Erasmus University Rotterdam, Tilburg University, and the University of Maryland (refer to Supplementary Material 5).

From 2010 to 2021, 30 countries have conducted related studies on neuroscience, and 10 countries have published at least five articles. Regarding published articles, almost all were conducted in a cooperative way, and there were many close cooperative relationships between universities in the United States and the Netherlands, which published more articles. The United States has the highest productivity in the field of neuroscience. A total of 61.9% (78 publications) were by Dutch authors. The Netherlands has the second-highest output in neuroscience, accounting for 18.3% (23 publications were from Dutch authors) (refer to Supplementary Materials 6, 7).

### Analysis of Methodological and Neuroscience Tools

In this study, the research tools and methods used in the resulting literature are categorized and summarized to better demonstrate the application of neuroscience methods in the field of marketing. We thusly concluded that neuromarketing has mainly been studied using eye tracking, fMRI, EEG/ERPs, and a combination of these tools. Supplementary Material 8 shows a summary of the research tool classification.

Eye tracking accounted for the highest proportion of tool used in neuromarketing research and is commonly used to study the impact of advertising graphics on consumers. For example, Mittal et al. (2021) studied individual differences in holistic and analytical thinking and plastic surgery preferences. Eye tracking was used to record the trajectory of women viewing neutral photographs of themselves. They found that focusing on specific body parts was associated with the desire to undergo relevant cosmetic surgery. Russell et al. (2017) tested the hypothesis that exposure to advertisements prior to TV episodes would increase attention to the location of product displays within the episodes and obtained conclusions consistent with this hypothesis. This tool has also been applied to studies on the impact of decisions regarding the nutritional value of food (Thomas et al., 2021).

However, the use of fMRI in this field cannot be ignored. It has been used mainly in studies on consumers’ physiological responses to marketing behaviors and in predicting consumer behavior through the relationship between the two variables and behavioral decisions. Of the 22 studies in which fMRI was used, half were related to branding and advertising. In contrast, Berns and Moore (2012) examined the possibility of using neuroimaging in smaller populations to predict the prevalence of culture. They found that the neural responses of the tested individuals could be applied to the general population. Although music sales cannot be predicted, they can be used to predict popularity. In contrast, Casado-Aranda et al. (2018) studied the relationship between risky and secure e-payments starting with neural means and analyzed the unconscious origin of consumers’ choice of payment system.

Using fMRI methods, this study found that EEGs/ERPs were used in neuromarketing in a direction similar to the main research themes. Barnett and Cerf (2017) used EEG to calculate the relative levels of neural similarity and cross-brain correlation (CBC) in audience movie trailers and demonstrated the role of these data in predicting future commercial data for movies. Pozharliev et al. (2015) examined brain responses to negative perceptions of luxury and basic brands alone or accompanied by another person. The results suggested that the presence of other people amplified the affective effects of the brand type. This is consistent with the results of De Vries et al. (2018), who used fMRI. Notably, only five studies used neurophysiological measures alone. They were mostly introductory experiments to new methods with no apparent thematic bias. Two of these studies used fNIRS to measure consumer-informed consumption behavior and consumer neural responses to different merchandise communication strategies at the PoS (Krampe et al., 2018; Çakir et al., 2018). Cerf et al. (2015) designed a series of experiments to explore the effects of food purchase/selection on food-related environmental odors in children and adults. Klesse et al. (2015) conducted five correlational experiments and found that speaking triggered more indulgent choices than manual expression patterns when consumers made requests, but not when using a foreign language. Cerf et al. (2015) presented a method for single-neuron recording in humans and discussed the relevance of this method to marketing and consumer behavior.

Finally, the remaining hybrid tools focused on the analysis of experimental data by combining two or more methods. This included fMRI and neurophysiological measures used to assess the role of “customer orientation” (CO) and “sales orientation” (SO) in personal selling from a biological perspective (Bagozzi et al., 2012). Moreover, EEG, skin conductance, and neurophysiological measure methods have been used to understand the impact of different ad placements and delivery tools on the mobile user experience (Clark et al., 2018). Varan et al. (2015) used data analysis from EEG, fMRI, facial coding, EMG, and biometrics studies and concluded that neuromarketing was not a reliable measure of advertising effectiveness. Devices, such as biometrics and skin conductance, are commonly found in hybrid tools.

Among the research methods used, the neuromarketing approach was mainly based on an experimental design, which accounted for 103 studies. Regarding other research, only 10 studies used a mixed research approach and six literature review studies (refer to Supplementary Material 9 for the Results). The review and methodology categories comprised six studies, as listed below. Rick (2011) collated and discussed three aspects of loss aversion. Chang (2017) discussed trends in advertising and marketing methodologies. Ramsøy (2019) explored the collation of research categories and concepts of neuroscience research on advertising effects and provided a framework for validating metrics based on neurosciences. Schmitt (2012) integrated empirical research and personal constructs into a framework model and provided criteria and methods for use. Venkatraman et al. (2015) proposed a method of mapping between cognitive processes and traditional marketing data based on neurosciences to improve consumer-product matching with traditional demographic methods. Yoon et al. (2012) proposed that neuroscience could shape future theories and models in consumer decision-making and that neuroscience methods could be used in decision-making research. These studies demonstrate that trends in the adoption of neuromarketing and research design guidelines continue to progress. However, the relevant developments only comprise the construction of research guidelines, frameworks, categories, and scopes of research.

### Keyword Analysis

**FIGURE 2:**
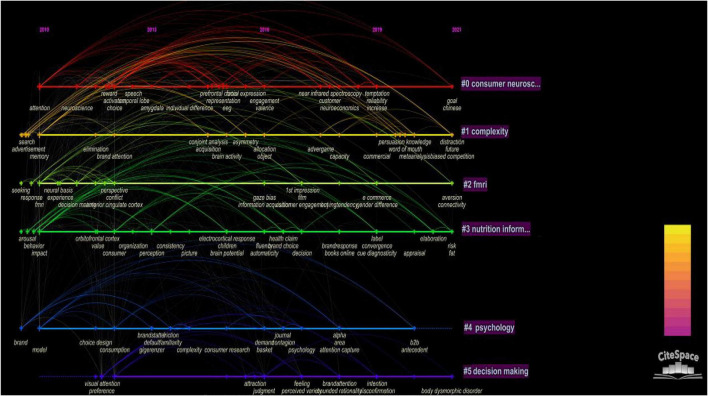
Timeline of the “Neuromarketing” cited network.

The timeline graph provides an overall view of the cluster timespan and how these clusters are connected. The results are shown in Supplementary Material 8. The keyword CC atlas can directly reflect the frequency of keywords in a research field. In this study, seven clusters were formed. The nodes in each row represent the keywords in each cluster and the links represent the relationships among the different keywords. In addition, the results showed that all seven clusters were closely connected. Cluster 0 was the largest because it contained the most articles. The continuous large nodes and extensive links in this cluster proved its activity, and the label of Cluster 0 represented the most noteworthy topic. Clusters 0 to 3 also had large nodes, indicating that they were relatively prominent topics in neuromarketing. In the CiteSpace interface, the keyword was taken as the node type and the time slice was set to 1. The G-index was used as the selection criterion and k = 25 was set. After running the software, the keyword CC atlas (Figure 2) was obtained. The atlas contained 233 nodes and 1195 connections, and the network density was 0.0442. Keywords with a frequency greater than or equal to 4, and centrality greater than or equal to 0.1, were listed (refer to Supplementary Material 8).

Cluster 0 was marked as “Consumer neuroscience,” meaning that the study of this cluster could be summarized as the study of consumer neuroscience. However, by reviewing the keywords of this classification, we found that the words with strong relationships included attention, neuroscience, activation, choice, reward, emotion, EEG, representation, neural response, amygdala, individual difference, cognition, and prefrontal cortex. These words reflected that the consumer neuroscience method analysis was mainly based on EEG tools, studying the prefrontal cortex and neuro response (Barnett and Cerf, 2017), and extracting the variables that influence consumers’ choices. The influence of emotion (mostly), attention, imagination, and other factors on consumers’ individual differences was focused on, and more importantly, the perspective of marketers (Venkatraman et al., 2012; Rampl et al., 2016).

Cluster 1 was marked as “Complexity,” meaning that the study of this cluster could be summarized as a study on complexity. However, by reviewing the keywords of this classification, words with strong relationships were found to include memory, attitude, advertisement, meta, analysis, and recognition. This popular vocabulary emphasizes the application of neuroscience in studies related to the effects of complex advertisements. For example, the study conducted by Pieters et al. (2010) found that advertisers affect consumers’ attention and attitude toward advertisements through the control of visual complexity to attract consumers’ attention. Others, such as Cian et al. (2014), used the effect of static vision to make consumers accustomed to it and induce consumers’ participation and attitude through dynamic images. Kuisma et al. (2010) used neuroscience technology to predict the value of advertisements and compared the unique preference (e.g., attitude) information between individuals and the whole population, especially from observing subjects’ reactions toward advertisements (e.g., eye movement).

Cluster 2 was marked as “fMRI,” meaning that the study in this cluster could be summarized as the study on fMRI. However, by reviewing the keywords of this classification, words with strong relationships were found to include fMRI, conflict, perspective, experience, decision-making, and response. These words show that the fMRI method was mainly used for study, including the evaluation of TV advertisements. Researchers have proposed a comprehensive program that combines a visual scale with fMRI to measure emotional responses to TV advertisements (Shen and Morris, 2016). Reimann et al. (2012) provided new insights into brand building by studying the psychological and neurophysiological mechanisms of how consumers relate to their favorite brands. Through three experimental studies, they found that emotional arousal decreased with the span of brand relationships. However, the degree of brand integration increased over time. Jai et al. (2021) analyzed consumers’ online purchase decisions based on the SOR theory and used fMRI image decisions to understand consumers’ purchase decisions and predict their purchasing behaviors by designing shoppers’ display products. According to the above studies, fMRI neuroscience tools can be used to analyze consumers’ behavior patterns in order to understand consumers’ purchasing behavior.

Cluster 3 was marked as “Nutrition information,” meaning that the study of this cluster could be summarized as the study on nutrition information. The main words included impact, perception, behavior, consumer, arousal, decision, health, and claims. These words reflect that this topic explored nutritional information. For example, Zou and Liu (2019) explored the influence of nutrition information on consumers’ decision-making to explore whether the label disclosing food ingredients by offline stores should be strengthened for the consumption of healthy food. Furthermore, Labban et al. (2021) studied the influence of commodity displays and healthy/unhealthy food on consumers’ purchase decisions. Some researchers have also discussed the influence of food health evaluations on consumers’ choice of packaged food through neuroscience experiments (Thomas et al., 2021). According to Cluster 3, the category of nutrition information was mainly discussed after 2019. Moreover, the issue of early food safety on consumers’ purchasing behavior was gradually changing from the traditional experimental and investigation research method (Baker, 1999; Ortega et al., 2014), to experimenting with and application of neuroscience.

Cluster 4 was marked as “Psychology,” meaning that the study of this cluster could be summarized as the study on psychology. However, by reviewing the keywords of this classification, words with strong relationships were found to include model, brand, and consumption. Unlike Cluster 0, this cluster no longer focused only on the advertisement itself. The study extended to the psychological influence of brand consumers (Albanese, 2015) and constructed a more accurate influence factor model by comparing neurophysiological responses in different advertisements (Venkatraman et al., 2015). The clustering direction mainly focuses on the influence of brand presentation on consumer psychology.

Cluster 5 was marked as “Decision making,” meaning that the study of this cluster could be summarized as the study on decision-making. The keywords of this classification mainly focused on the influence of the coherent application of eye tracking to consumer decision-making. The core popular words included brain, bias, visual attention, preference, judgment, and eye tracking. In the application of clustering, visual attention and eye tracking were mainly used to elucidate consumers’ subjective initiative consciousness and to understand the influence of consumers’ preferences on their judgment when they respond to brands (Milosavljevic et al., 2012; Telpaz et al., 2015; Yang et al., 2018; Shi and Trusov, 2021).

**FIGURE 3:**
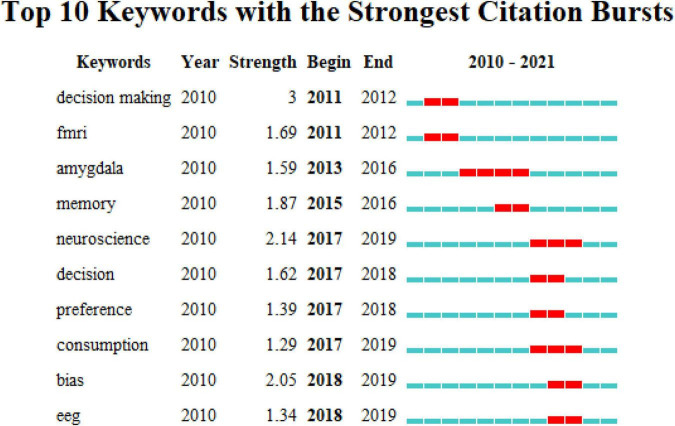
The top 10 keywords with the strongest citation bursts from 2010 to 2021.

In addition, all data from 2000 to 2021 were comprehensively analyzed, and all keywords were extracted to detect any keyword outburst. Keyword outbursts show the frequent occurrence of any keyword in a specific period. This information not only shows the evolution of research hotspots over time but also shows the research trend in recent years and may indicate future developmental trends (Mou et al., 2019). The ten keyword outbursts identified by the analysis results are shown in Figure 3. Combining the evolutionary path diagram with the keywords with high outburst intensity, we concluded the following key meanings. First, in 2010, there were many studies on neuroscience, among which keywords, such as fMRI, memory, information, attention, and model, received extensive attention at that time. This indicates that the studies focused on the relationship between human physiological response and consumers (Aribarg and Schwartz, 2020; Kuisma et al., 2010; Reimann et al., 2012). With the deepening of experiments, an increasing number of research tools have shifted to other tools such as EEG. Especially after 2017, in-depth studies were conducted on the relationship between substances in the brain and specific actions or reactions of people, which was more targeted (Barnett and Cerf, 2017; Barwise et al., 2020). In addition, words, such as decisions and preferences, have been discussed in more topics since 2017. For example, Çakir et al. (2018) investigated the neural correlates of purchase behavior using fNIRS. Simultaneously, the influence of the job satisfaction of salespeople was further discussed (Bagozzi and Verbeke, 2020). Therefore, for application in neuromarketing, the analysis methods of neuroscience gradually became diversified. These comprise not only the early eye tracking or fMRI methods but also extended to neurophysiology methods in recent years to predict the physiological status of consumers’ EEG for decision-making analysis of marketing behavior.

## Discussion and Implications

### Research Discussion

According to our research on neuromarketing articles from 2010 to 2021 in journals with an AJG rating of 3 and above, neuroscience equipment can be applied to marketing experimental research and has profound development prospects (de Oliveira and Giraldi, 2017). The analysis results showed the following conclusions and effects:

Regarding publication trends, two journals in the field of marketing, i.e., the *Journal of Consumer Psychology* and the *Journal of Marketing Research*, continue to publish special issues of neuromarketing. These are also two leading journals in the ABS 4*marketing field. This indicates that the marketing field is also beginning to take a neuroscience approach seriously. Specifically, these two journals are among the top five in terms of publications as well as citation status (average citation rates of 50.27 and 80.54%, respectively). In particular, the *Journal of Consumer Psychology* is among the top six most-cited journals for the period of 2010–2021, with three papers by Schmitt (2012); Reimann et al. (2010), and Milosavljevic et al. (2012). This journal has the highest number of highly cited papers. The journal with the highest average citation rate per paper is the *Journal of Marketing*. It is also a top journal with an ABS rating scale of 4*. This indicates the journal’s impact. Notably, Pieters et al.’s (2010) study was the most cited journal after 2010. The number of citations in this study was 217. The impact of excluding complex ad-hocness on experiments proposed by this study is also paving the way for subsequent studies on the indicative nature of advertising experiments. Regarding trends, the main journals receiving articles in neuromarketing continue to have a bias toward consumer psychology.

In contrast, regarding institutional and national publication status, the United States has the highest number of publications, followed by the Netherlands. However, the collaboration networks among Michigan University, Erasmus University Rotterdam, Tilburg University, and the University of Maryland are reflected in the top journals of the *Journal of Marketing Research* and *Journal of Marketing*, respectively. It has collaborated on topics such as advertising effectiveness (Aribarg et al., 2010; Pieters et al., 2010; Teixeira et al., 2012; Smidts et al., 2014; Pozharliev et al., 2015) and consumer behavioral decision-making (Bagozzi et al., 2012; Yoon et al., 2012; Pozharliev et al., 2015; Bagozzi and Verbeke, 2020). It can be concluded that neuromarketing is not deeply collaborative among countries. At present, only a few research institutions have strong collaborative relationships.

The cluster analysis results regarding domain user keywords indicated that the most dominant clusters were “Consumer neuroscience,” “Complexity,” “fMRI,” “Nutrition information,” “Psychology,” and “Decision making.” Research hotspots focused on consumer decision analysis (Yang et al., 2018; Shi and Trusov, 2021), hybrid neuroscience tools (e.g., applying EEG, fMRI, fNIRS, neurophysiology, and so forth) (Clark et al., 2018; Jai et al., 2021); complexity (Pieters et al., 2010), and nutritional information (Labban et al., 2021; Thomas et al., 2021) as leading research frontiers. To further understand the time-varying hot topics, this study analyzed the keyword progression for keyword emergence. The results revealed a high level of interest in the relationship between human physiological responses and consumers from 2010 to 2015. After 2017, there has been a multifaceted discussion of consumer satisfaction, which is more focused on or elicited from an indirect correlation experimental approach. The keywords derived from either the cluster analysis or keyword-emergent analysis methods were consistent. These keyword clusters closely matched the elements of the neurosciences of human feedback to stimuli. Simultaneously, it reflects the importance of the consumer, a key object in the marketing field. It shows a clear difference in tendency between the two phases, which may stem from the updating of the experimental equipment and methods. It was originally introduced to marketing in the field of neuroscience, when the research was more akin to the field of medicine. With the introduction of new equipment and the accumulation of antecedent studies, researchers have delved deeper or expanded their research horizons.

### Implications for Academic Research

The nature of neuromarketing studies relates to consumers. This topic increases in importance as the field of marketing science evolves. Through the collation of bibliometric coupling, keyword emergence, and the manual reading of research articles, including the use of research methods and neuroscience tools, this study presents the following promising themes to offer future researchers for further discussion.

First, the integration of multiple neuroscience tools or methods is a future research trend, based on the embodiment of research results. Gradual diversification in the use of neuroscience tools has been observed. For example, studies by Clement et al. (2017), Grewal et al. (2018), Bellman et al. (2019), and Bagozzi and Verbeke (2020) have not only used neuroscience experiments, but also have incorporated survey research parties or multiple neuroscience experimental tools. Therefore, the impact of consumer behavioral decisions can be discussed through hybrid studies in the future, which can more accurately predict and explore the impact of consumer behavioral decisions.

Regarding the topic direction, researchers can approach it from two perspectives, namely, nutrition information and decision-making. The former focuses on food safety information, particularly on the issue of product information disclosure. Related studies include those of Labban et al. (2021) and Thomas et al. (2021). These studies have focused on the orientation of consumer behavioral decisions extended by traditional food safety (Ortega et al., 2014). In a follow-up study, the analysis can be conducted in terms of the impact of food passports or origin history on consumer purchasing decisions and through neuroscience experiments. Within the topic of consumer behavioral decision-making, most research in neuromarketing continues to discuss experiments related to consumer decision-making, including studies such as Barwise et al. (2020), Huang et al. (2021), and Jia et al. (2022). However, in the field of marketing, the design of marketing plans by employes or decision analysis by supervisors in the corporate sector is also important research topics that influence the effectiveness of marketing behavior drivers. Therefore, further research could be conducted in the neurosciences to investigate issues related to marketing decisions in companies. This research can also address the gaps in traditional business leaders’ cognitive reactions, decision-making, and emotional expressions that are not easily measured and observed when making marketing decisions, to elucidate information processing and decision-making at the corporate level.

### Research Limitation

As the keyword nodes presented by CiteSpace can only appear qualitatively, there are some limitations to this study. From a quantitative perspective, the value represented by the nodes comes from the number of times the keyword appears in the entire literature dataset. If the software could weigh the number of times the keywords and author order appeared in the literature, then the study would be more convincing. In contrast, the data analysis refers to ABS 3-star journals in the marketing field as the source of data acquisition. *Behavioral and Brain Sciences*, *Frontiers in Neuroscience*, *Frontiers in Behavioral Neuroscience*, and *BMC Neuroscience* have all included the analyzes of consumer behavioral decisions. There are also many neuromarketing-related topics in marketing seminars organized by the American Marketing Association and other marketing societies. None of the studies in this section have been considered. This may also be a limitation of this study.

## Data Availability Statement

The original contributions presented in this study are included in the article/Supplementary Material, further inquiries can be directed to the corresponding authors.

## Author Contributions

ZZ, YS, and YJ designed the research, provided guidance throughout the entire research process, collected the references, did the literature analysis, and wrote the manuscript. KJ and C-LL helped with translation and offered modification suggestions. C-LL and XL participated in the literature collection, analysis, and organization. All authors contributed to the article and approved the submitted version.

## Conflict of Interest

The authors declare that the research was conducted in the absence of any commercial or financial relationships that could be construed as a potential conflict of interest.

## Publisher’s Note

All claims expressed in this article are solely those of the authors and do not necessarily represent those of their affiliated organizations, or those of the publisher, the editors and the reviewers. Any product that may be evaluated in this article, or claim that may be made by its manufacturer, is not guaranteed or endorsed by the publisher.
