# Supplementary material for: Bibliometric-Based Evaluation of the Neuromarketing Research Trend: 2010–2021
Source: Front Psychol. 2022 Aug 2;13:872468. doi: 10.3389/fpsyg.2022.872468 (PMC9380815; doi:10.3389/fpsyg.2022.872468)
Supplement: Supplementary file 1 [file Data_Sheet_1.docx]

Supplementary Material 1:


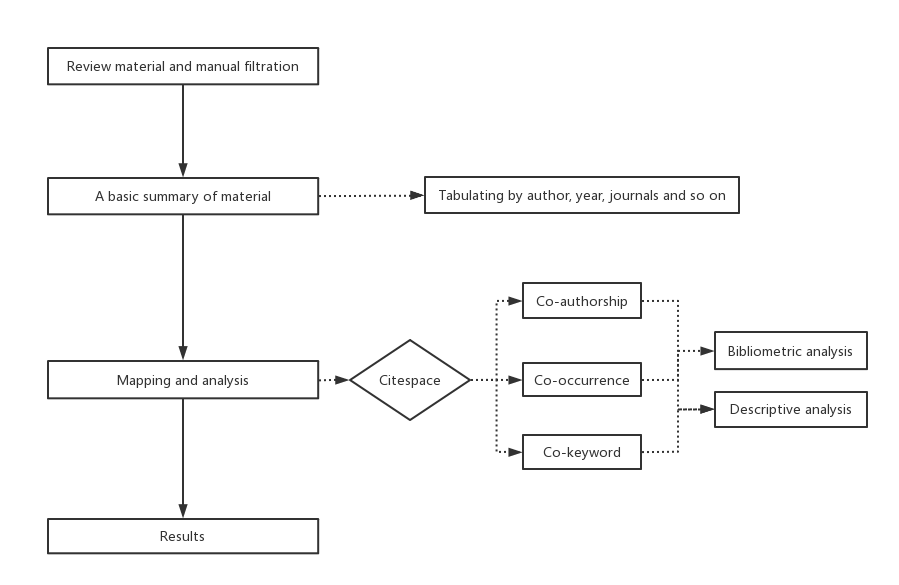


Process framework

Supplementary Material 2:


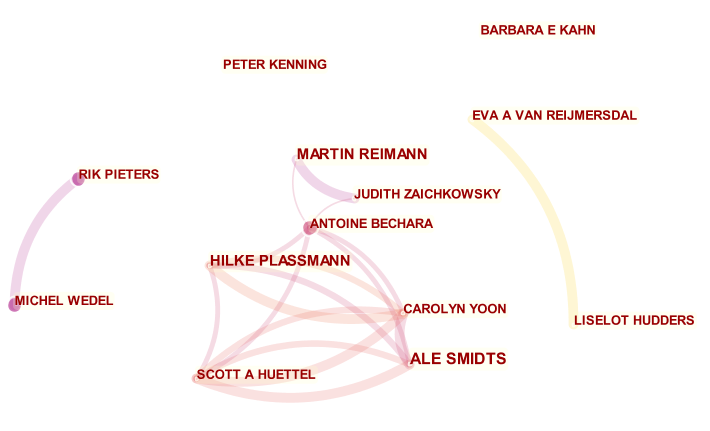


Author cooperation network with more than three posts

Supplementary Material 3: The authors with more than 3 articles and their affiliated institutions

| **Rank** | **Authors** | **Affiliation** | **Documents** |
| --- | --- | --- | --- |
| 1 | Ale Smidts | Erasmus University Rotterdamersity | 4 |
| 2 | Martin Reimann | University of Southern California | 4 |
| 3 | Michel Wedel | University of Maryland | 3 |
| 4 | Eva A Van Reijmersdal | University of Amsterdam | 3 |
| 5 | Liselot Hudders | Ghent University | 3 |
| 6 | Rik Pieters | Tilburg Universityersity | 3 |
| 7 | Antoine Bechara | University of Southern California | 3 |
| 8 | Judith Zaichkowsky | Copenhagen Business Schoolool | 3 |
| 9 | Barbara E Kahn | University of Pennsylvania | 3 |

Supplementary Material 4: Most cited publications**.**

| **Rank** | **Author(Year)** | **Title** | **Citations** |
| --- | --- | --- | --- |
| 1 | Pieters et al. (2010) | The Stopping Power of Advertising: Measures and Effects of Visual Complexity | 217 |
| 2 | Schmitt (2012) | The consumer psychology of brands | 210 |
| 3 | Reimann et al. (2010) | Aesthetic package design: A behavioral, neural, and psychological investigation | 209 |
| 4 | Venkatraman et al. (2015) | Predicting Advertising Success Beyond Traditional Measures: New Insights from Neurophysiological Methods and Market Response Modeling | 191 |
| 5 | Milosavljevic et al. (2012) | Relative visual saliency differences induce sizable bias in consumer choice | 173 |
| 6 | Teixeira et al. (2012) | Emotion-Induced Engagement in Internet Video Advertisements | 159 |

Supplementary Material 5: Institutions with more than 5 articles published

| Ranking | Organizations | Documents |
| --- | --- | --- |
| 1 | Michigan university | 11 |
| 2 | Erasmus University Rotterdam | 8 |
| 3 | Duke University | 6 |
| 4 | Tilburg University | 5 |
| 5 | Copenhagen Business School | 5 |
| 6 | Columbia University | 5 |
| 7 | Ghent University | 5 |
| 8 | University of Maryland | 5 |
| 9 | University of Pennsylvania | 5 |

Supplementary Material 6: Countries with more than 5 articles posted

| Ranking | Countries | Documents | Citations |
| --- | --- | --- | --- |
| 1 | USA | 73 | 2960 |
| 2 | Netherlands | 22 | 996 |
| 3 | Australia | 11 | 209 |
| 4 | France | 10 | 243 |
| 5 | Canada | 10 | 399 |
| 6 | England | 9 | 265 |
| 7 | People's Republic of China | 9 | 84 |
| 8 | Denmark | 7 | 404 |
| 9 | Germany | 7 | 397 |
| 10 | Belgium | 5 | 35 |

Supplementary Material 7:


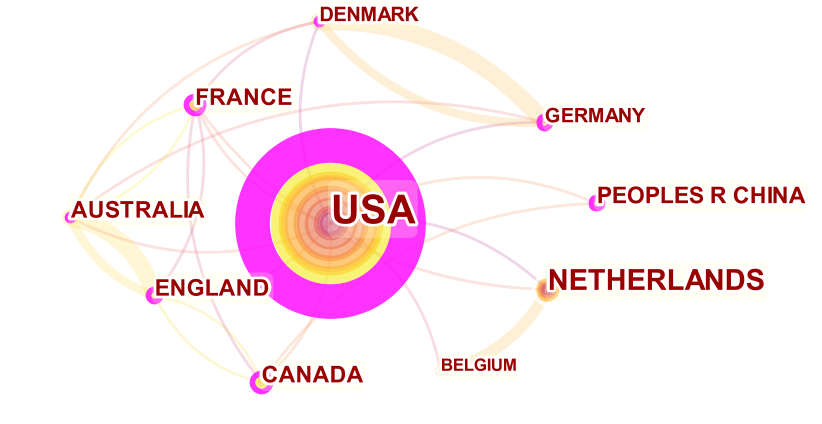


The national cooperation network with more than four published articles

Supplementary Material 8:


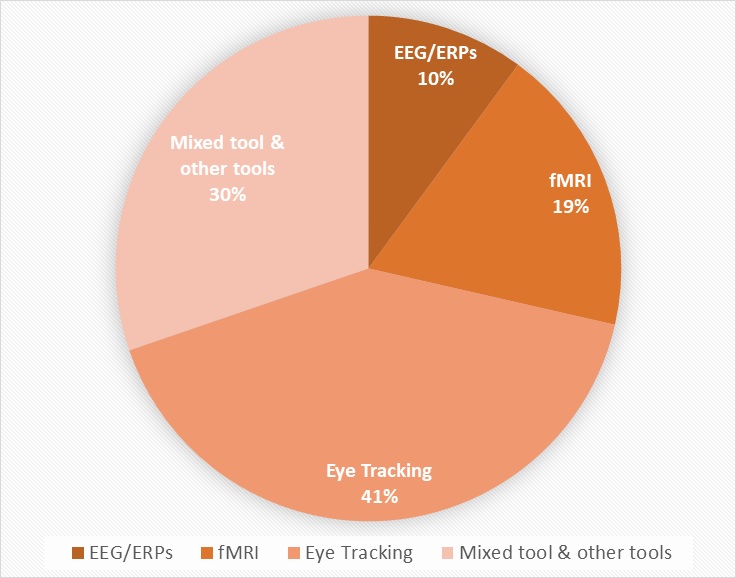


Analysis of neuroscience tools from 2010–2021

Supplementary Material 9:


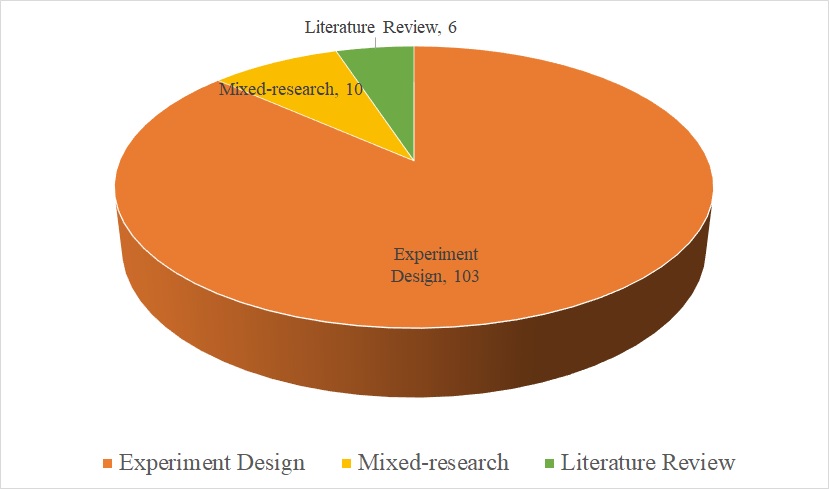


Analysis of methodologies from 2010–2021
